# Editorial: Hypertension in obese women: gender-specific challenges and solutions

**DOI:** 10.3389/fendo.2025.1558754

**Published:** 2025-02-04

**Authors:** Santiago Cuevas, Jussara M. Do Carmo, Rodrigo O. Marañón

**Affiliations:** ^1^ Physiopathology of the Inflammation and Oxidative Stress Lab, Molecular Inflammation Group, BioMedical Research Institute of Murcia (IMIB-Pascual Parrilla), El Palmar, Spain; ^2^ Department of Physiology and Biophysics, University of Mississippi Medical Center, Jackson, MS, United States; ^3^ Sex Differences in Cardiovascular, Metabolic, and Renal Diseases Research Lab, Faculty of Medicine, National University of Tucuman, Centro Científico Tecnológico (CCT), Consejo Nacional de Investigaciones Científicas y Técnicas (CONICET), Tucuman, Argentina

**Keywords:** obesity, women, hypertension, preecclampsia, research

Hypertension is a prevalent condition affecting millions worldwide, with a notable incidence among obese women. This population faces unique gender-specific challenges that exacerbate the risks associated with hypertension. Factors such as hormonal fluctuations, increased body mass index, and lifestyle choices contribute to the complex mechanisms between obesity and hypertension. By identifying and addressing these gender-specific challenges, it can help to improve outcomes for obese women with hypertension, and contributing to reduced morbidity and mortality in this vulnerable population. This Research Topic provides an overview of the Research Topic, highlighting its significance and summarizing the contributions made by the included articles.

## Significance of the Research Topic

Obesity and elevated Body Mass Index (BMI) in pregnant women are significant risk factors for complications such as gestational hypertension, preeclampsia, and gestational diabetes. It has been evidenced that obesity is associated with an increased risk of adverse outcomes, including preterm birth, cesarean delivery, and fetal overgrowth (macrosomia). Also, a higher BMI contributes to maternal inflammation, which can exacerbate vascular dysfunction and increase the risk of pregnancy-related hypertension. In obese women, adipose tissue produces elevated levels of pro-inflammatory cytokines (e.g., TNF-α, IL-6) and decreases anti-inflammatory adipokines (e.g., adiponectin). Furthermore, the chronic low-grade inflammation disrupts placental function, affecting nutrient and oxygen delivery to the fetus and increasing the risk of fetal growth abnormalities. Inflammation and endothelial dysfunction induced by obesity can impair blood pressure regulation and contribute to hypertensive disorders. Placental inflammation might trigger immune responses that further escalate maternal systemic inflammation, increasing the risk of adverse pregnancy outcomes. A better understanding of the mechanisms involved in the axis of obesity, blood pressure regulation, sex differences, and inflammation are essential to determine new strategies to mitigate these risks including pre-conception weight management, anti-inflammatory dietary interventions, and careful monitoring of maternal health during pregnancy.

## Contributions of the Research Topic

Obesity and elevated BMI in pregnant women are significant risk factors for gestational complications. Obese women have an increased risk of hypertension, cardiovascular diseases, strokes, and kidney complications. These factors, are presumably influenced by increased vascular resistance, activation of the renin-angiotensin-aldosterone system (RAAS), chronic inflammation, and insulin resistance. Premenopausal women experience some protection against hypertension due to estrogen; however, hormonal changes such as reduced estrogen after menopause and adipocyte dysfunction further exacerbate the condition. Obese individuals also produce higher levels of leptin, which can overstimulate the sympathetic nervous system, leading to elevated blood pressure. Additionally, unhealthy lifestyle factors, including high-sodium diets and physical inactivity, significantly amplify these risks and have a profound impact in such cases.

Thus, all evidence indicate that obesity is a critical modifiable risk factor for hypertension, and targeted interventions focused on weight management can substantially improve health outcomes in hypertensive obese women. Weight loss and lifestyle modifications, combined with appropriate medical treatment, are essential for effective management. However, it is crucial to determine the molecular mechanisms involved in this pathological process to identify suitable medications that can significantly benefit these patients.

This Research Topic encompasses a wide range of issues, including the molecular mechanisms linking obesity and high blood pressure, the interplay between metabolism, sex differences, and inflammatory pathways. An important study included in our Research Topic explored the relationship between pre-pregnancy BMI and the risk of developing gestational hypertension (GH). Elevated pre-pregnancy BMI is linked to a higher risk of GH, especially in pregnancies achieved through assisted reproductive technologies such as frozen-thawed embryo transfer (FET), which have a greater risk compared to fresh embryo transfers. A retrospective cohort including 7,502 women achieved singleton pregnancies after FET. The overall incidence of GH was 6.15% in participants were categorized into normal BMI, overweight, and obese group. The risk of GH was significantly higher in the obese group (15.55%) compared to the overweight (8.26%) and normal BMI groups (4.68%). For each unit increase in pre-pregnancy BMI, the risk of GH increased by 16.4% until a turning point at a BMI of 26.8 kg/m². Women with overweight and obesity also showed higher rates of gestational diabetes, preterm births, and cesarean deliveries. Neonates born to these mothers had higher birth weights, increased risks of macrosomia (birth weight ≥4,000 g), and low birth weight (<2,500 g) compared to those born to mothers with normal BMI. All these data evidence that pre-pregnancy overweight and obesity are significant risk factors for GH after FET. Maintaining a normal BMI before FET can substantially reduce this risk (Fan et al.).

Zhou et al. also develops a risk prediction model for preeclampsia (PE) based on pre-pregnancy body mass index (BMI) and peripheral blood biomarkers: placental growth factor (PLGF), decorin (DCN), lactate dehydrogenase (LDH), and uric acid (UA). Pre-pregnancy BMI, PLGF, DCN, LDH, and UA are significant factors influencing PE development. The model, visualized using a nomogram, can effectively predict PE risk and guide early interventions highlight the importance of a multifactorial approach for accurate PE prediction and prevention. Following similar approach to determine the casual pathways involved in PE, Tan et al. performed an important analysis including 267,242 individuals, with a focus on European ancestry. Ancestry included 2,355 patients with PE. The study identified strong genetic associations linking an increased risk of pre-eclampsia with factors such as hyperthyroidism, BMI, type 2 diabetes and elevated serum uric acid levels as expected. Conversely, no significant causal associations were found with gestational diabetes, total cholesterol, sleep duration and bone mineral density, suggesting areas for further investigation. A notable finding was the causal association between systemic lupus erythematosus and increased risk of PE, highlighting the role of immune and inflammatory responses. Additionally other factors of specific metabolic, lipid, immune, lifestyle and bone metabolism factors may causal impact in PE development, supporting that a multidimensional approach to better understand and manage PE, paving the way for future research to develop targeted preventive and therapeutic strategies.

Additionally factors have been study by Turner et al. that may have significant impact in obesity. There are emerging areas of study that may shed light in the mechanisms underlying sex-specific shaping of the HPA sensitivity in response to early life stress. Adverse childhood experiences (ACEs) shape the adrenal-adipose tissue axis, predisposing women to obesity and related cardio metabolic risks. Early life stress (ELS) disrupts neuroendocrine pathways, especially the hypothalamic-pituitary-adrenal (HPA) axis, leading to heightened susceptibility to obesity and metabolic syndrome in females which show an increased vulnerability due to hormonal interactions, such as aldosterone’s role in fat storage and inflammation. Animal models of maternal separation provide insights into these mechanisms, revealing that ELS primes the HPA axis for heightened stress responses, promoting adipose tissue dysregulation. Obesity in females exposed to ELS is linked to increased leptin and aldosterone production, exacerbating metabolic dysfunction. All these evidences suggest a need for targeted therapeutics addressing, these unique pathways in women to mitigate long-term health consequences of ACEs.

In order to find the most relevant molecular pathways involved in metabolic syndrome development, Hamby et al. investigates the effects of the absence of ASIC2 and βENaC proteins on metabolic syndrome caused by a high-fat diet (HFD) in mice. Mice lacking ASIC2 and βENaC showed reduced weight gain, lower fasting blood glucose, improved glucose tolerance, reduced liver fat accumulation, and better lipid profiles compared to wild-type mice. Female mice demonstrated greater protection, with less liver fat, reduced insulin resistance, and lower macrophage infiltration in the liver. These findings suggest that targeting ASIC2 and βENaC pathways could offer new therapeutic strategies for managing metabolic syndrome and obesity-related diseases and their pharmacological regulation may have significant impact of these patients.

Obesity and elevated BMI are critical risk factors for gestational complications, including GH and PE particularly in pregnancies achieved through assisted reproductive technologies. Emerging research highlights the complex interplay of hormonal, genetic, metabolic, and lifestyle factors, emphasizing the importance of maintaining a healthy pre-pregnancy BMI and identifying molecular pathways for targeted interventions. Studies on early life stress, hormonal interactions, and specific genetic markers, such as the role of ASIC2 and βENaC, underscore the need for a multifactorial approach to predict, prevent, and manage obesity-related complications effectively.

## Conclusions

The multifaceted risks associated with obesity and elevated BMI, particularly in pregnant women, highlighting their significant role in gestational complications such as hypertension, preeclampsia, and adverse neonatal outcomes. It underscores the importance of maintaining a healthy pre-pregnancy BMI and leveraging predictive tools, including genetic and biomarker-based models, to mitigate these risks. Furthermore, research into early life stress, hormonal interactions, and novel molecular pathways, such as ASIC2 and βENaC, are needed to provide promising avenues for targeted therapeutics and prevention strategies, aiming to address the complex interplay of metabolic, genetic, and environmental factors in managing obesity-related complications.

